# Prävention in der Tumornachsorge

**DOI:** 10.1007/s00106-026-01756-y

**Published:** 2026-03-31

**Authors:** Matthias Santer, Charles Schmit, Lukas Schmutzler, Daniel Dejaco, Benedikt Hofauer

**Affiliations:** Universitätsklinik für Hals‑, Nasen‑, Ohrenheilkunde, Medizinische Universität Innsbruck, Anichstr. 35, 6020 Innsbruck, Österreich

**Keywords:** Kopf-Hals-Tumoren, Prävention, Plattenepithelkarzinom, Behandlungsergebnis, Krankheitsmanagement, Head and neck neoplasms, Prevention, Squamous cell carcinoma, Treatment outcome, Disease management

## Abstract

**Hintergrund:**

Mit den steigenden Remissions- und 5‑Jahres-Überlebens-Raten erhält die präventionsorientierte Nachsorge von Kopf-Hals-Tumoren eine zunehmende Relevanz. Neben der onkologischen Kontrolle rücken präventive Maßnahmen zur Vermeidung von Rezidiven, Zweitkarzinomen sowie funktionellen Langzeitkomplikationen verstärkt in den Mittelpunkt.

**Zielsetzung:**

Ziel dieser Übersichtsarbeit war die Analyse präventiver Strategien in der Tumornachsorge von Patientinnen und Patienten mit Kopf-Hals-Tumoren.

**Material und Methoden:**

Es erfolgte eine narrative Literaturrecherche in PubMed zur präventionsorientierten Nachsorge bei Kopf-Hals-Tumoren. Ergänzend wurden nationale und internationale Leitlinien berücksichtigt.

**Ergebnisse:**

Strukturierte Nachsorgeprogramme ermöglichen die frühzeitige Detektion von Lokalrezidiven und therapieassoziierten Zweitneoplasien. Molekulare Verfahren wie die Liquid Biopsy bieten vielversprechende Perspektiven für eine individualisierte, risikoadaptierte Nachsorge, bedürfen jedoch weiterer prospektiver Validierung. Lebensstilinterventionen, insbesondere Rauch- und Alkoholkarenz, sind mit einer Reduktion des Rezidiv- und Zweittumorrisikos assoziiert. Die HPV-Impfung wirkt präventiv im Rahmen der Primärprävention HPV-assoziierter Tumoren. Eine frühzeitige funktionelle Rehabilitation in den Bereichen Stimme, Schlucken, Ernährung und Mobilität trägt wesentlich zur Prävention dauerhafter Funktionsdefizite bei.

**Schlussfolgerung:**

Eine strukturierte, interdisziplinäre Tumornachsorge ermöglicht eine frühzeitige Rezidiverkennung und verbessert funktionelle Ergebnisse sowie die Lebensqualität. Innovative Ansätze bedürfen jedoch weiterer klinischer Validierung.

## Abstract (en)

**Background:**

In light of increasing remission and 5‑year survival rates, prevention-orientated follow-up care of patients after head and neck cancer is becoming increasingly relevant. In addition to oncologic surveillance, measures aimed at preventing recurrent, second primaries, and long-term functional sequalae are gaining importance.

**Objective:**

The aim of this review is to analyze preventive strategies in the follow-up care of patients with head and neck cancer.

**Materials and methods:**

A narrative literature review was conducted in PubMed, focusing on prevention-oriented follow-up care in head and neck cancer. In addition, national and international guidelines (Association of the Scientific Medical Societies in Germany, AWMF; National Comprehensive Cancer Network, NCCN) were considered.

**Results:**

Structured follow-up programs enable early detection of local recurrences and treatment-associated secondary neoplasms. Molecular approaches such as liquid biopsy offer promising perspectives for individualized, risk-adapted follow-up care but require further prospective validation. Lifestyle interventions, particularly smoking and alcohol cessation, are associated with a reduction in the risk of recurrence and second primary tumors. Human papillomavirus (HPV) vaccination exerts a preventive effect within the framework of primary prevention of HPV-associated tumors. Early functional rehabilitation in the domains of voice, swallowing, nutrition, and mobility substantially contributes to the prevention of persistent functional deficits.

**Conclusion:**

Structured interdisciplinary follow-up care enables early detection of recurrence and improves functional outcomes as well as quality of life. However, innovative approaches require further clinical validation.

Die Nachsorge bei Kopf-Hals-Tumoren vereint onkologische Tertiärprävention zur frühzeitigen Erkennung von Rezidiven und Zweitneoplasien mit funktioneller Primärprävention. Ziel ist die Verbesserung der Langzeitprognose sowie der Erhalt von Stimme, Schluckfunktion, Ernährung und psychosozialer Stabilität. Dies erfordert einen individualisierten, interdisziplinären Nachsorgeansatz. Die Behandlung von Kopf-Hals-Tumoren erfordert aufgrund der komplexen topografischen Anatomie und der zentralen funktionellen Bedeutung der betroffenen Strukturen ein hohes Maß an interdisziplinärer Expertise in Diagnostik, Therapie und Nachsorge [1]. Letztere stellt einen essenziellen Pfeiler des onkologischen Gesamtkonzepts dar und verfolgt mehrere komplementäre Zielsetzungen ab Bestimmung des Remissionsstatus im Rahmen eines Restagings. So dient die Nachsorge der frühzeitigen Identifikation von Lokalrezidiven und therapieassoziierten Zweitmalignomen. Hierdurch leistet sie einen substanziellen Beitrag zur Senkung der tumorassoziierten Morbidität und zur Verbesserung der Gesamtprognose [2]. Mit den kontinuierlichen Fortschritten in der Therapie und der damit einhergehenden Zunahme der 5‑Jahres-Überlebensrate, wie beispielsweise bei fortgeschrittenen Kopf-Hals-Tumoren durch Immuntherapie, gewinnt der funktionelle Aspekt der Nachsorge zunehmend an Bedeutung. [1, 3, 4]. Der Erhalt beziehungsweise die Wiederherstellung essenzieller Funktionen, wie der Phonation oder des Schluckens, ist von zentraler Relevanz für die langfristige Lebensqualität der Patientinnen und Patienten. Frühzeitige präventive Interventionen sind daher integraler Bestandteil aktueller Nachsorgekonzepte [5]. Ein individualisierter, multiprofessioneller Nachsorgeansatz ist daher unerlässlich, um sowohl die onkologische Kontrolle als auch die funktionelle Rehabilitation zu gewährleisten und den komplexen Langzeitbedürfnissen dieser Patientengruppe umfassend zu begegnen [6, 7]. Aktuelle Leitlinien (NCCN, AWMF) fokussieren in der Nachsorge primär auf die onkologische Verlaufskontrolle (klinische Untersuchung, Bildgebung, Symptomkontrolle). Präventive bzw. funktionell-präventive Maßnahmen werden überwiegend nur randständig oder implizit adressiert, sodass daraus bislang nur begrenzt konkrete, operationalisierbare Empfehlungen ableitbar sind [8–14].

## Material und Methoden

Es erfolgte eine explorative Literaturrecherche in PubMed zur präventionsorientierten Tumornachsorge bei Patientinnen und Patienten mit Kopf-Hals-Tumoren. Es wurden dabei Artikel eingeschlossen, die in den letzten 20 Jahren veröffentlicht wurden. Ergänzend wurden einschlägige nationale und internationale Leitlinien (AWMF, NCCN) berücksichtigt. Eingeschlossen wurden relevante Originalarbeiten, Übersichtsarbeiten und Leitliniendokumente zur Nachsorgestruktur.

## Tertiärprävention in der Tumornachsorge

Die Tertiärprävention beginnt mit dem Abschluss der kurativen Primärtherapie, also dem Erreichen einer Vollremission, und stellt einen zentralen Baustein der onkologischen Langzeitbetreuung dar [15]. Ziel ist die frühzeitige Identifikation von Lokalrezidiven und therapieassoziierten Zweitneoplasien, um durch rechtzeitige therapeutische Intervention die kurative Perspektive möglichst zu wahren und die funktionelle Integrität zu erhalten. Eine strukturierte, risikoadaptierte und leitlinienbasierte Nachsorge bildet hierbei das Fundament einer präventionsorientierten Tumorkontrolle [6].

In der klinischen Routine umfasst diese regelmäßige HNO-ärztliche Untersuchungen, inklusive Inspektion, Palpation und Endoskopie zur gezielten Beurteilung der Primärlokalisation und typischer Rezidivareale. Bei limitiert einsehbaren Regionen kommen Panendoskopien selektiv zum Einsatz. Die radiologische Bildgebung ist essenziell für die Detektion besonders subklinischer Rezidive. In standardisierten Intervallen durchgeführte Bildgebungen wie Computertomographie, Magnetresonanztomographie, Sonographie sowie Positronenemissionstomographie ermöglichen die frühzeitige Detektion morphologisch unklarer Befunde und spielen eine zentrale Rolle in der Bestimmung des Remissionsstatus [1, 6]. Zunehmend werden KI-gestützte Verfahren und Radiomics in die Bildanalyse der Nachsorge integriert. Erste Studien zeigen Potenzial für automatisierte Mustererkennung und Risikostratifizierung, was Sensitivität und Effizienz der Nachsorge künftig erhöhen könnte. Derzeit befinden sich diese Ansätze jedoch überwiegend noch in einem explorativen Stadium, da heterogene Protokolle, aufwendige Segmentierung, kleine Kohorten und fehlende externe Validierung die klinische Routineanwendung limitieren [16, 17].

Im Fall eines diagnostizierten Rezidivs schließt sich die sog. Salvage-Therapie an, deren Erfolg maßgeblich vom frühzeitigen Erkennen und der präzisen Ausdehnungsdiagnostik abhängt. Salvage-Eingriffe erfordern in der Regel eine detaillierte interdisziplinäre Planung unter Berücksichtigung anatomischer Komplexitäten, bereits erfolgter Vorbehandlungen und patientenspezifischer Faktoren. Funktionelle Zielgrößen, Komorbiditäten, psychosoziale Ressourcen und Lebensqualität sind integraler Bestandteil der Entscheidungsfindung. Präventive Maßnahmen zur Vermeidung von Wundheilungsstörungen, Fistelbildung oder funktioneller Einbußen sollten frühzeitig implementiert werden, wie eine präoperative Ernährung, lokale Lappenplastiken wie ein Pektoralis-major-Lappen zur Fistelprophylaxe bei Laryngektomien oder, um funktionelle Defizite zu antizipieren, ein freier Radialis-Lappen zur Rekonstruktion des Weichgaumens, um die nasale Regurgitation zu limitieren [8–10, 18, 19].

**Abb. 1:**
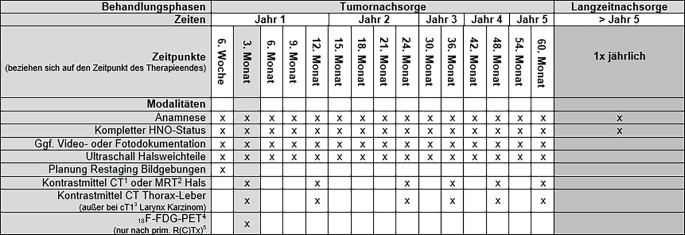
Strukturierte Tumornachsorge der Univ.-Klinik für Hals‑, Nasen- und Ohrenheilkunde – Kopf-Hals-Chirurgie Innsbruck mit implementierten präventiven Maßnahmen. ^1^ Computertomographie; ^2^ Magnetresonanztomographie; ^3^ TNM 8. Auflage, ^4 18^F‑Fluordesoxyglukose-Positronenemissionstomographie; ^5^ primäre Radio(chemo)therapie. Jede Spalte unter „Tumornachsorge“ repräsentiert einen standardisierten Nachsorgetermin. Im Rahmen der Tumornachsorge implementierte präventive Maßnahmen umfassen: Rezidivfrüherkennung (lokal, regional, fernmetastatisch), Screening auf Zweitmalignome, Mangelernährung, Schmerzchronifizierung, psychoonkologische Belastung, funktionelle Einschränkungen (Dysphagie, Dysphonie, Bewegungsapparat), therapieassoziierte Komorbiditäten. Förderung sozialer und beruflicher Reintegration sowie strukturierter Lebensstilinterventionen (Rauchstopp, Alkoholkarenz, ernährungsmedizinische Beratung, Schleimhautpflege)

Ein weiteres Ziel der präventionsorientierten Nachsorge ist die frühzeitige Erkennung von strahleninduzierten Zweitmalignomen, da ein Großteil der Patientinnen und Patienten mit Kopf-Hals-Tumoren im Rahmen der Primärtherapie bestrahlt wird. Zweitneoplasien, welche potenziell strahleninduziert sind, treten metachron im bestrahlten Gewebe in bis zu 10 % der Fälle auf [20]. Die kumulative Exposition gegenüber ionisierender Strahlung begünstigt über DNA-Schäden und oxidativen Stress eine maligne Transformation. Besonders häufig betroffen sind die Schleimhäute des Oropharynx, Hypopharynx und Larynx sowie sekundär exponierte Organe wie die Schilddrüse. Mit der Einführung der intensitätsmodulierten Radiotherapie (IMRT) konnte die Toxizität gesenkt werden ([21, 22]). Exemplarisch ist die Tumornachsorge der Univ.-Klinik für Hals‑, Nasen- und Ohrenheilkunde – Kopf-Hals-Chirurgie Innsbruck mit den implementierten präventiven Maßnahmen dargestellt (Abb. 1)

### Lifestyle-Modifikation

Veränderbare Lebensstilfaktoren zählen zu den effektivsten Hebeln tertiärpräventiver Nachsorgestrategien. Der anhaltende Konsum von Tabak und Alkohol erhöht nachweislich das Risiko für Rezidive und Zweitneoplasien bei Kopf-Hals-Malignomen. Die Nachsorge bietet ein strukturiertes, zeitlich fokussiertes Interventionsfenster, um gesundheitsriskantes Verhalten zu modifizieren. Bis zu 80 % der Kopf-Hals-Tumoren sind mit einem Tabakkonsum assoziiert [24]. Ein evidenzbasierter, multimodaler Ansatz, bestehend aus verhaltenstherapeutischer Intervention, Nikotinersatz und ggf. medikamentöser Unterstützung, ist besonders wirksam. Erfolgsentscheidend sind zusätzlich die strukturierte Zuweisung zu Entwöhnungsprogrammen und digital unterstützte Angebote. Die NCCN geht in einer gesonderten Leitlinie dazu ein [25, 26]. Die kumulative Inzidenz für Zweitkarzinome beträgt insgesamt etwa 3–7 % pro Jahr, mit einer 20-Jahres-Kumulativrate von bis zu 36 % und ist somit mehr als doppelt so hoch bei persistierendem Nikotinabusus [20, 27]. Auch ein Alkoholentzug, idealerweise im Sinne einer vollständigen, permanenten Abstinenz, sollte Bestandteil der präventiven Nachsorge sein, da dieser die Entstehung von Plattenepithelkarzinomen begünstigen kann – unter anderem über seinen karzinogenen Metaboliten Acetaldehyd sowie durch eine erhöhte Schleimhautpermeabilität, die die Aufnahme krebserregender Substanzen wie Nitrosamine erleichtert. Zusätzlich wirken Alkohol und Nikotin synergistisch und potenzieren sich in ihrem karzinogenen Potenzial [28]. Weitere vermeidbare Risikofaktoren im Rahmen der präventiven Nachsorge umfassen den Konsum von Kautabak (z. B. Betelnussprodukte), mangelhafte orale Hygiene sowie die berufliche Exposition gegenüber karzinogenen Substanzen wie Asbest oder Formaldehyd. Eine sorgfältige arbeitsmedizinische Anamnese und die enge Zusammenarbeit mit Arbeitsmedizin und Rehabilitationsdiensten sind für die risikoadaptierte Beratung und Prävention essenziell [29].

### Molekulare Nachsorge

Die molekulare Nachsorge markiert einen Paradigmenwechsel in der Tertiärprävention. Ziel ist der Nachweis molekularer Residualerkrankung oder früher Rezidive vor einer klinischen oder bildgebenden Manifestation. Im Zentrum steht die Liquid Biopsy, bei der zirkulierende Tumor-DNA (ctDNA) aus Blut, Speichel oder Urin analysiert wird. Persistierende oder ansteigende ctDNA-Spiegel können als Surrogatmarker für eine subklinische Tumorlast dienen. Studien zeigen, dass freie HPV-ctDNA im Blut bei Oropharynxkarzinom-Patientinnen und -Patienten mit einem signifikant erhöhten Rezidivrisiko assoziiert ist. In einzelnen Fällen wurde Tumorprogression bis zu sechs Monate vor der Bildgebung detektiert. Moderne Verfahren wie digitale PCR, NGS sowie DNA-Methylierungsanalysen verbessern Sensitivität und Spezifität der Verfahren. Der Einsatz KI-basierter Algorithmen zur Mustererkennung molekularer Daten wird derzeit intensiv untersucht. Trotz dieser Fortschritte bleibt die klinische Integration vorerst experimentell [30, 31].

### HPV-Prävention

HPV-assoziierte Oropharynxkarzinome weisen im Vergleich zu HPV-negativen Tumoren eine günstigere Prognose auf. Die aktuellen Leitlinien empfehlen die HPV-Impfung ausschließlich zur Primärprävention; ein Nutzen in der Tumornachsorge oder bei manifestem Oropharynxkarzinom ist bislang nicht belegt. Zwar reduziert die Impfung die Prävalenz oropharyngealer Hochrisiko-HPV-Infektionen deutlich, belastbare prospektive Daten zur direkten Senkung der OPSCC-Inzidenz fehlen jedoch bislang, sodass hierfür derzeit keine Empfehlung ausgesprochen werden kann [8, 11].

### Telemedizin als präventives Nachsorgeinstrument

Telemedizinische Nachsorgekonzepte stellen eine vielversprechende Ergänzung zu klassischen Versorgungsstrukturen dar. Video-Visiten und appbasiertes Symptom-Monitoring ermöglichen eine frühzeitige Erkennung funktioneller Einschränkungen (z. B. Dysphagie, Dysphonie) sowie psychischer Belastungen. Durch die Möglichkeit einer hochfrequenten Erfassung solcher Symptome können zudem Lokalrezidive oder Zweitmalignome früher erkannt werden. Digitale Werkzeuge fördern darüber hinaus die Gesundheitskompetenz und Adhärenz der Patientinnen und Patienten, verbessern den Informationsfluss, bauen Barrieren ab und stärken die Eigenverantwortung. In der Breitenversorgung können telemedizinische Module dazu beitragen, personelle und infrastrukturelle Ressourcen gezielt und effizient einzusetzen [32, 33].

### Stimmrehabilitation

Eine frühzeitige Stimmtherapie kann ein essenzieller Bestandteil der funktionellen Prävention, insbesondere bei Patientinnen und Patienten mit laryngealen Tumoren oder nach Laryngektomie, sein. Der Verlust der physiologischen Stimmfunktion beeinträchtigt nicht nur die kommunikative Teilhabe, sondern hat auch erhebliche psychosoziale Folgen bis hin zur sozialen Isolation. Eine frühzeitige logopädisch-phoniatrische Mitbetreuung ist daher von zentraler Bedeutung, um chronische Dysphonien zu vermeiden und ggf. bei einer Dysphonie individuell geeignete Rehabilitationsstrategien einzuleiten. Nach totaler Laryngektomie gilt die Versorgung mit einer Stimmprothese (z. B. Provox®, Atos Medical AB, Hörby, Schweden) als Goldstandard der Ersatzstimmgebung. Dadurch gelingt bei über 60 % der Betroffenen eine suffiziente Rehabilitation [34]. Voraussetzung für den Erfolg sind eine strukturierte Schulung, die individuelle Anpassung sowie eine kontinuierliche Nachsorge [35]. Ergänzend können elektrolaryngeale oder ösophageale Ersatzstimmen zum Einsatz kommen. Der Einsatz erfolgt idealerweise im Rahmen eines interdisziplinären, präventiv orientierten phoniatrisch-onkologischen Rehabilitationskonzepts und trägt wesentlich zur Verbesserung der Lebensqualität bei [34].

### Dysphagiemanagement und Ernährungstherapie

Dysphagien gehören zu den häufigsten funktionellen Folgeerscheinungen nach Therapie von Kopf-Hals-Tumoren, mit den höchsten Raten bei fortgeschrittenen Tumoren des Oropharynx, Hypopharynx und des zervikalen Ösophagus. Sie beeinträchtigen nicht nur die Lebensqualität, sondern auch die Prognose durch ein erhöhtes Risiko für Aspiration, Mangelernährung und Pneumonie. Studien berichten über Prävalenzen von mehr als 50 % [36, 37]. Eine frühzeitige Evaluation ist daher von essenzieller Bedeutung. Interdisziplinäre Konzepte, bestehend aus HNO, Phoniatrie, Logopädie und Diätologie, ermöglichen eine differenzierte Diagnostik, beispielsweise mittels einer FEES zu standardisierten Intervallen und damit verbunden der Einleitung gezielter Therapien. Dazu zählen kompensatorische Schlucktechniken, myofunktionelle Übungen, thermisch-taktile Stimulation und bei inadäquater Kompensation die temporäre bis permanente enterale Ernährung über PEG [37–39]. Das Monitoring einer suffizienten Ernährung ist ein Grundpfeiler der Tumornachsorge. Das Screening sollte neben der Gewichtsentwicklung auch standardisierte Laborparameter inkl. Entzündungsmarker (z. B. CRP) sowie „nutrition impact symptoms“ erfassen. Entzündungsassoziierte Mangelernährung und Sarkopenie sind bei Kopf-Hals-Tumoren häufig und mit mehr Toxizität und schlechterer Prognose assoziiert, weshalb eine frühzeitige Ernährungstherapie indiziert ist [40]. Neben Dysphagien beeinträchtigen Mukositis, Xerostomie und Dysgeusie infolge von Radiatio die orale Nahrungsaufnahme und führen häufig zu Mangelernährung oder tumorassoziierter Kachexie [41]. Die frühzeitige Integration diätologischer Expertise ist unerlässlich, um individuelle Ernährungspläne zu erstellen und einer negativen Energiebilanz vorzubeugen. In Risikosituationen sollte eine enterale Ernährung mittels PEG frühzeitig diskutiert werden. Eine PEG sollte nicht routinemäßig prophylaktisch, sondern risikostratifiziert erwogen werden (z. B. bei signifikanter Dysphagie/Aspiration, relevantem Gewichtsverlust oder absehbar unzureichender oraler Zufuhr unter [Chemo-]Radiotherapie). Die Entscheidung sollte interdisziplinär erfolgen und mit früher schluckspezifischer Therapie sowie regelmäßiger Verlaufskontrolle kombiniert werden. Eine frühzeitige PEG kann zwar Mangelernährung und Therapieabbrüche reduzieren, ist jedoch mit Sondenkomplikationen und potenziell erhöhter Langzeitabhängigkeit assoziiert [40–42].

### Bewegungstherapie und physikalische Therapie

Körperliche Inaktivität begünstigt Dekonditionierung, muskuläre Dysbalance und Fatigue – besonders nach ausgedehnten operativen Eingriffen oder Radiochemotherapie. Eine Bewegungstherapie kann daher ein zentrales präventives Instrument in der onkologischen Nachsorge sein. Individuell angepasste Rehabilitationsprogramme, kombiniert mit physiotherapeutischen und motivational-psychologischen Elementen, können nicht nur die körperliche Leistungsfähigkeit steigern, sondern auch Immunkompetenz und psychisches Wohlbefinden stabilisieren. Gruppenangebote, digitale Trainingsplattformen und strukturierte Heimprogramme erhöhen die Adhärenz und unterstützen die soziale Reintegration [43]. Die radiogen bedingte Fibrosierung sowie Lymphödeme stellen schwerwiegende funktionelle Spätfolgen der Strahlentherapie dar und können unbehandelt zu Immobilität, Schmerzen und dauerhafter Funktionseinschränkung führen. Präventive physikalische Maßnahmen, insbesondere Lymphdrainage, Manualtherapie, Dehnübungen und thermische Anwendungen, sind essenziell zur Vermeidung von Bewegungseinschränkungen. So kann eine frühzeitige Therapieinitialisierung die Fibrosierung limitieren und die Lebensqualität langfristig erhalten [44].

### Psychosoziale Prävention und soziale Reintegration

Die psychosoziale Belastung durch eine Kopf-Hals-Krebserkrankung ist erheblich. Angststörungen, Depressionen und soziale Isolation sind häufige Folgeerscheinungen und wirken sich negativ auf Therapieadhärenz und Lebensqualität aus. Psychoonkologische Interventionen sind daher kein optionales, sondern ein integrales Element der präventiven Nachsorge. So sind Depressionen mit einem signifikant reduzierten Gesamtüberleben assoziiert (Hazard Ratio 0,868; 95 %-Konfidenzintervall 0,819–0,921; *p* < 0,001) [45]. Rehabilitationszentren mit spezialisierten Programmen bieten neben der medizinischen Versorgung auch psychosoziale Unterstützung und berufliche Wiedereingliederung. Kommunikationsrehabilitation, Selbsthilfegruppen sowie digitale Austauschplattformen tragen dazu bei, soziale Isolation zu vermeiden und die Selbstwirksamkeit der Betroffenen zu stärken [43].

## Ausblick

Die Nachsorge bei Kopf-Hals-Tumoren gewinnt durch steigende Remissions- und Überlebensraten zunehmend an Bedeutung. Neben der Tumorkontrolle stehen präventive Maßnahmen zur Vermeidung von Rezidiven, Zweitkarzinomen und funktionellen Spätfolgen im Fokus. Ein strukturiertes Nachsorgemanagement, Lebensstilinterventionen (z. B. Rauch- und Alkoholkarenz), HPV-Impfung sowie digitale Instrumente wie Telemonitoring wirken präventiv bzw. verbessern die Früherkennung und Adhärenz. Frühzeitige funktionelle Maßnahmen unterstützen zudem wichtige Funktionen wie Phonation und reduzieren die Dysphagierate.

## Fazit für die Praxis

- Die Prävention in der Nachsorge von Kopf-Hals-Tumoren gewinnt durch steigende Remissions- und Überlebensraten zunehmend an Bedeutung.
- Strukturierte und risikoadaptierte Nachsorgeprogramme verbessern die frühzeitige Erkennung von Lokalrezidiven und Zweittumoren.
- Lebensstilinterventionen wie Tabak- und Alkoholkarenz sind ein essenzieller Bestandteil der Prävention.
- Molekulare Verfahren bieten vielversprechende Ansätze zur frühzeitigen Rezidivdiagnostik.
- Die HPV-Impfung kann die Inzidenz HPV-induzierter Oropharynxkarzinome senken.
- Interdisziplinäre und digital unterstützte Konzepte fördern Adhärenz, Autonomie und Lebensqualität.
- Funktionelle Prävention umfasst eine frühzeitige Unterstützung bei Stimme, Schlucken, Ernährung, Bewegung sowie psychischer Stabilisierung.

## Data Availability

Für diese Studie wurden keine öffentlich zugänglichen Datensätze generiert. Relevante Daten sind im Artikel enthalten.
